# Analysis of Triclosan-Selected *Salmonella enterica* Mutants of Eight Serovars Revealed Increased Aminoglycoside Susceptibility and Reduced Growth Rates

**DOI:** 10.1371/journal.pone.0078310

**Published:** 2013-10-18

**Authors:** Ulrike Rensch, Guenter Klein, Corinna Kehrenberg

**Affiliations:** Institute of Food Quality and Food Safety, University of Veterinary Medicine Hannover, Foundation, Hannover, Germany; Institut National de la Recherche Agronomique, France

## Abstract

The biocide triclosan (TRC) is used in a wide range of household, personal care, veterinary, industrial and medical products to control microbial growth. This extended use raises concerns about a possible association between the application of triclosan and the development of antibiotic resistance. In the present study we determined triclosan mutant prevention concentrations (MPC) for *Salmonella enterica* isolates of eight serovars and investigated selected mutants for their mechanisms mediating decreased susceptibility to triclosan. MPC_TRC_ values were 8 - 64-fold higher than MIC values and ranged between 1 - 16 µg/ml. The frequencies at which mutants were selected varied between 1.3 x 10^-10^ - 9.9 x 10^-11^. Even if MIC values of mutants decreased by 3-7 dilution steps in the presence of the efflux pump inhibitor Phe-Arg-β-naphtylamide, only minor changes were observed in the expression of genes encoding efflux components or regulators, indicating that neither the major multidrug efflux pump AcrAB-TolC nor AcrEF are up-regulated in triclosan-selected mutants. Nucleotide sequence comparisons confirmed the absence of alterations in the regulatory regions *acrRA, soxRS, marORAB, acrSE* and *ramRA* of selected mutants. Single bp and deduced Gly_93_→Val amino acid exchanges were present in *fabI*, the target gene of triclosan, starting from a concentration of 1 µg/ml TRC used for MPC determinations. The *fabI* genes were up to 12.4-fold up-regulated. Complementation experiments confirmed the contribution of Gly_93_→Val exchanges and *fabI* overexpression to decreased triclosan susceptibility. MIC values of mutants compared to parent strains were even equal or resulted in a more susceptible phenotype (1-2 dilution steps) for the aminoglycoside antibiotics kanamycin and gentamicin as well as for the biocide chlorhexidine. Growth rates of selected mutants were significantly lower and hence, might partly explain the rare occurrence of *Salmonella* field isolates exhibiting decreased susceptibility to triclosan.

## Introduction

Biocides are broadly used to control microbial growth and, thus play an essential role in preventing the spread of infectious agents and disease [1]. In food animal production, the consumption of biocides has been increased during recent years. This has been in response not only to consumer demands for healthy, minimally processed food without preservatives or antimicrobial agents but also to ensure food safety and outbreak containment [2,3]. In this food animal environment, biocides are used for cleaning and disinfecting farm buildings or abattoirs to improve hygiene and to reduce microbial loads. In addition, they act as preservatives in animal feed or in animal-derived products (e.g. semen, eggs) or can be used for animal skin spot-on applications such as teat dips [1]. In contrast to most antimicrobial agents, biocides affect multiple target sites of the bacterial cells and therefore, decreased susceptibility to biocides is thought to occur more rarely [4]. Despite this, tolerance of bacteria to biocides is documented for most classes, and increasing percentages of tolerant strains might contribute to the survival of zoonotic pathogens in the food chain and might facilitate the emergence of bacterial persisters [3,5]. In addition, concerns have been raised about a possible association between the overuse of biocides and the development of antimicrobial resistance. Nevertheless, it is not clear if these results achieved under laboratory conditions are transferable to a comparable situation in a natural environment [6,7]. 

Triclosan (TRC), a halogenated biphenyl ether, is used in Europe, North America and Asia in a wide variety of products and has become the most commonly used antibacterial agent in the United States [6,8]. It is used as an antibacterial adjunct in medical devices such as surgical suture material or hand soaps [9] and is also found in a variety of personal care and household products including soaps, deodorants, toothpastes, antiseptic-creams, plastics and functional clothing [10]. It has been used for more than 40 years as a single active ingredient or as a component in biocide formulations and, as a consequence, triclosan is commonly detected in the aquatic environment [1,8]. At low concentrations, triclosan inhibits the enoyl-acyl carrier protein FabI, an enzyme required for the fatty acid synthesis of bacteria, by mimicking the natural substrate [11,12]. At higher concentrations, a nonspecific effect on cell membranes and the interference with proteins or with the bacterial lipid synthesis have been described [6,12]. Increased tolerance of bacteria to triclosan can develop through multiple mechanisms such as modifications in the target site or over-production of FabI, changes in the membrane permeability of cells, or enhanced multidrug efflux pump activity [8,13]. In *Stenotrophomonas* and *Pseudomonas*, triclosan binds to efflux pump repressor proteins and, subsequently, leads to a release of the repressor from regulatory regions and to an up-regulation of downstream targets [7,14]. The de-repression of multidrug efflux systems, which are able to export a wide range of compounds, including triclosan, out of the cell contributes to a less susceptible antibiotic phenotype as shown for several bacterial species, e.g. *Pseudomonas aeruginosa, Stenotrophomonas maltophilia, Escherichia coli* and *Salmonella enterica* [7,15–17]. Nevertheless, inconsistent findings have been reported and other studies failed to demonstrate cross-resistance between biocide tolerance and antibiotic resistance [6,18,19].

*Salmonella enterica* is a major cause of enteric illness worldwide. Human disease is often associated with the consumption of contaminated food, with poultry products often acting as vehicles [20]. Due to increasing percentages of *Salmonella* isolates resistant to commonly used antibiotics, the treatment of severe infections is becoming more difficult [2]. In a previous comparative analysis, testing of 375 avian *Salmonella* isolates revealed no increase in triclosan MIC values of isolates collected during different time periods [21]. Nonetheless, enhanced expression of multidrug efflux pumps in response to biocide usage is a matter of concern although the mediated levels of antimicrobial resistance are relatively low [22]. The major efflux pump in *Salmonella*, AcrAB-TolC, is involved not only in antimicrobial resistance, but also in virulence [23]. Its substrate spectrum includes antibiotics, quaternary ammonium compounds, acridines as well as triclosan and the functional activity of AcrAB can be complemented by another efflux system, AcrEF [2]. In Enterobacteriaceae, expression of AcrAB-TolC is known to be regulated at various levels including global regulators such as MarA, SoxS and RamA and a local repressor, AcrR [24,25]. In quinolone-resistant salmonellae, structural changes in regulatory genes such as *ramA* and *soxS* have been identified which modulate efflux pump expression [24,25]. Furthermore, it has recently been shown that several toxic compounds such as dequalinium induce *ramA* expression as well [26]. Nevertheless, very little is known about alterations in the relevant regions of multidrug efflux regulators following biocide exposure. 

It was the aim of the present study (i) to determine the level of concentrations of triclosan required to inhibit the emergence of mutants, (ii) to investigate mechanisms mediating decreased triclosan susceptibility in selected mutants of various serovars and (iii) to determine changes in the antimicrobial susceptibility and in the growth rates of generated mutants.

## Materials and Methods

### Bacterial strains and determination of mutant prevention concentrations

Eight *Salmonella enterica* subsp. *enterica* isolates of avian origin comprising the serovars Enteritidis, Hadar, Infantis, Livingstone, Paratyphi B, Saintpaul, Typhimurium and Virchow were used as parent strains in this study. The isolates were collected between 2002 and 2008 during a surveillance study of non-typhoidal salmonellae in Germany. Isolates were cultivated overnight at 37°C on Luria Bertani (LB) agar plates or in LB broth (Oxoid, Wesel, Germany). Following Randall et al. 2004, the lowest concentration inhibiting the emergence of mutants from ≥10^10^ cells was defined as the mutant prevention concentration (MPC) [27,28]. For MPC determination, an overnight culture was centrifuged and cells were concentrated in 5 ml 0.9 % NaCl. Ten 100 µl aliquots of the cell suspension were plated on agar plates supplemented with triclosan in a concentration of 1 - 256 x the MIC value. Plates were incubated at 37°C. After 24h and 48h, the MPC_24h_ and MPC_48h_ were determined as the lowest concentration that inhibited the growth of any mutant. In addition, the mutant frequency was calculated by dividing the number of mutants grown at a specific concentration of triclosan by the number of CFU/ml inoculum. The molecular mechanisms of decreased susceptibility to triclosan were investigated for selected mutants.

### Susceptibility testing and efflux pump inhibition

To assess MIC values of the biocides triclosan, acriflavine, benzalkonium chloride and chlorhexidine (purchased from Sigma Aldrich, Munich, Germany and AppliChem, Darmstadt, Germany) susceptibility testing was performed by the broth macrodilution method and as previously described, *S. Enteritidis* 7112 was used as the wild-type control [21]. Procedures regarding inoculum density, growth medium and incubation times and conditions were in accordance with the Clinical and Laboratory Standards Institute (CLSI) guidelines [29]. The susceptibility of isolates to the antimicrobial agents tetracycline, chlorampheniol, florfenicol, gentamicin, kanamycin and ciprofloxacin was investigated by broth macrodilution. Performance and interpretations followed the recommendations given in the CLSI document VET01-A4 [29]. For quality control purposes, *E.coli* ATCC 25922 was used as a reference strain. For all selected mutant strains, MICs were determined in the presence and absence of the efflux pump inhibitor phenylalanyl-arginyl-β-naphthylamide (PaβN), (Sigma-Aldrich, Munich, Germany) that inhibits RND efflux pumps such as AcrAB-TolC. PaβN was used in a concentration corresponding to 0.25 x the MIC of the inhibitor [30].

### Stability of the triclosan tolerant phenotype and growth kinetics of mutant strains

The phenotypic stability of decreased susceptibility to triclosan was investigated by MIC determination after a daily subculture of generated mutants (five CFU per serovar) for 10 successive days on agar plates without any supplement. Susceptibility testing to biocides and antibiotics was repeated after the serial passage. Changes in the growth kinetic between *S. Enteritidis*, *S*. Paratyphi B and S. Saintpaul parent strains and their corresponding triclosan tolerant mutants were assessed. For this, an overnight culture was diluted in a 10-fold dilution series (up to 10^-4^) and 1 ml of the diluted culture were inoculated into 99 ml fresh LB broth to obtain approximately 5 x 10^2^ CFU. Cells were incubated at 37°C with shaking (140 rpm) for 19 h and optical density readings at 600 nm and numbers of CFU/ml were recorded every hour. Every growth kinetic experiment was performed three times independently.

### DNA preparation, PCR amplification and sequence analysis

Total genomic DNA was extracted by using the Dneasy Blood and Tissue Kit (Qiagen, Hilden, Germany). The gene *fabI* and, for a choice of isolates, the regions *acrRA*, *soxRS*, *marORAB*, *acrSE* and *ramRA*, encoding gene products known to be involved in the regulation of AcrAB-TolC, were amplified by PCR as previously described [20,24,31,32]. Amplification products were sequenced on both strands (Eurofins MWG, Ebersberg, Germany) and the nucleotide and deduced amino acid sequences were compared for parent strains and their triclosan mutants by using the DNAMAN software (Lynnon BioSoft, Quebec, Canada). In addition, macrorestriction analysis was conducted as previously described to confirm the origin of the mutants [33].

### Expression analysis of efflux pump genes and *fabI*

Quantitative real-time PCR analysis (qRT-PCR) was used to assess the expression of efflux pump genes *acrA*, *tolC* and *acrF*, regulatory genes *marA*, *ramA* and *soxS* and the gene encoding FabI. Overnight cultures of parent strains and mutants were grown to the mid-logarithmic phase and the total RNA was extracted by using the RNeasy Mini Kit (Qiagen, Hilden, Germany) according to the manufacturer´s instructions. DNase digestion (RNase-Free DNase Set, Qiagen) was done to eliminate contaminating DNA. Extracted RNA was quantified for yield using a Biophotometer (Eppendorf, Hamburg, Germany). Amounts of 0.5 µg RNA were reverse-transcribed into cDNA by using the QuantiTect Reverse Transcription Kit (Qiagen). Reverse-transcription minus controls were included to confirm the absence of genomic DNA. qRT-PCR was performed in a LightCycler 480 II instrument (Roche, Mannheim, Germany) with each specific primer pair as previously described [25,34,35] by using the LightCycler 480 SYBR Green 1 Master Kit (Roche). Amplifications were performed with an initial step of 5 min at 95°C, followed by 45 cycles of 10 s at 95°C, 10 s at 60°C and 10 s at 72°C. The gene *gyrB* was included as a reference gene [25]. All qRT-PCRs were performed in three independent experiments. The relative expression software tool (REST) was used for calculating and analyzing qRT-PCR results [36].

### Cloning and transformation experiments

The entire gene *fabI* plus 157 and 141 bp in the up- and downstream regions were amplified by PCR from genomic DNA of *S. Paratyphi* B parent (*fabI* original) and the respective mutant strain (*fabI* Gly_93_→Val). For this, previously described primers and the Taq DNA polymerase (Invitrogen, Karlsruhe, Germany) were used [20]. Amplicons from both strains were cloned into vector TOPO pCR2.1 (Invitrogen, Karlsruhe, Germany) and transformed into recipient strains *E. coli* TOP10, *S. Typhimurium* LT2 and the S. Livingstone field isolate 4. The *fabI* nucleotide sequences of all recombinant plasmids were checked for mutations and confirmed by sequence analysis and sequence alignments. *E. coli* TOP10 and *Salmonella* recipients harbouring the confirmed recombinant vector plasmids were tested for their susceptibility to triclosan, chloramphenicol and tetracycline and for control purposes for their susceptibility to the biocides chlorhexidine, benzalkonium chloride and acriflavine. Susceptibility testing was performed in the presence and absence of the efflux pump inhibitor PaβN. qRT-PCR experiments assessed the gene expression of *fabI*.

### Statistical analysis

Data were calculated and interpreted using the software GraphPad Prism6 (GraphPad Software, La Jolla, USA). For comparison of growth curves, the non-parametric Mann-Whitney-U-Test was used and differences were considered significant when p < 0.05.

## Results and Discussion

### Bacterial susceptibility of parent strains and MPCs of triclosan

Among the *Salmonella* field isolates, MIC values of triclosan (MIC_TRC_) varied between 0.125 - 0.5 µg/ml, these results being within the range recently reported for avian salmonellae in Germany [21]. For the biocides acriflavine, benzalkonium chloride and chlorhexidine, the MICs were 32 to 128 µg/ml, 32 µg/ml and 1 to 8 µg/ml, respectively. Susceptibility testing to antimicrobial agents revealed that two isolates of serovars Enteritidis and Typhimurium were susceptible to all antimicrobials tested, whereas the isolates of serovars Paratyphi B, Livingstone, Infantis and Hadar were resistant to tetracycline. Isolates of serovars Saintpaul and Virchow showed combined resistance to gentamicin and kanamycin (S. Saintpaul) or chloramphenicol and tetracycline (S. Virchow). MICs of florfenicol varied between 2 to 8 µg/ml. For all *Salmonella* serovars, mutants showing decreased susceptibility to triclosan were selected easily overnight and concentrations between 1 to 16 µg/ml triclosan were necessary to inhibit the emergence of any mutant (MPC value) after 24 or 48 h of incubation. The MIC/MPC ratios were calculated and ranged between 8 and 64. The frequency of mutation to decreased triclosan susceptibility was in the range of 1.3 x 10^-10^ to 9.9 x 10^-11^ for all serovars and thus, seems to be slightly lower than the frequency to mutation calculated after ciprofloxacin selection [17,37]. For each serovar, one mutant was selected from an agar plate supplemented with triclosan in a concentration of one dilution step below the MPC for further analysis.

### Efflux activity of selected mutants

After four serial passages on LB agar plates supplemented with triclosan, selected mutants exhibited 64- to >512-fold higher MIC values of triclosan than the parent strains (Table 1). Growth in the presence of 0.25-fold MIC of the inhibitor PaβN decreased the MIC_TRC_ values by 3 to 7 dilution steps, a finding that may result from an inhibition of the multidrug efflux pumps AcrAB-TolC or AcrEF, previously identified to be involved in triclosan and/or biocide tolerance of *Salmonella* [2,38]. As efflux inhibition pointed towards enhanced efflux activity, the level of expression of genes *acrA*, *tolC, acrF*, *ramA*, *soxS* and *marA*, encoding efflux components or regulators, was assessed by comparative qRT-PCR. In contrast to previous investigations of *Salmonella* mutants obtained after ciprofloxacin exposure or exposure to biocide formulations [2,25], only minor increases in *acrA, tolC* and *acrF* gene expression levels were observed, indicating that neither AcrAB-TolC nor AcrEF are up-regulated in the triclosan mutants. The changes ranged from 0.9- to 1.9-fold (for *acrA*), 0.8- to 1.1-fold (for *tolC*) and from 0.8- to 1.2-fold (for *acrF*). Divergent findings were reported by Karatzas et al. 2007, who detected an association of triclosan exposure with overexpression of the AcrAB-TolC efflux pump gene *acrB*, suggesting that *Salmonella* strains or exposure conditions may affect the results [4]. Slightly higher increases in gene expression were observed for *marA* (up to 2.5-fold) or *ramA* (up to 3.4-fold), whereas similar to the response to tertiary amine compounds or mixtures of aldehydes and quaternary ammonium compounds [2], a minor down-regulation of *soxS* (up to 0.3-fold) was determined (Table 2). Sequencing of *S. Paratyphi* B and S. Saintpaul *acrRA*, *soxRS*, *marORAB*, *acrSE* and *ramRA* regions and comparisons between parent strains and their respective mutants did not detect any sequence alteration that might cause *ramA* or *marA* up-regulation. 

**Table 1:** Mutant prevention concentrations (MPCs) and MICs of triclosan for *Salmonella* parent strains and generated mutants.

	**Analysis of *Salmonella* field isolates^a^**							**Characteristics of selected mutants**	
***Salmonella* isolates**	**Years of isolation**	**MIC_TRC_ (µg/ml)**	**MPC_TRC_^b^ (µg/ml)**		**MPC/MIC**		**Frequency of mutant selection**	**MIC (µg/ml)**	
			**24 h**	**48 h**	**24 h**	**48 h**		**TRC**	**TRC in the presence of 0.25 x MIC PAβN**
*S.* Enteritidis 92	2006	0.25	4	4	16	16	5.4 x 10^-11^	32	1
*S.* Hadar 5	2002	0.125	1	2	8	16	4.4 x 10^-11^	16	2
*S.* Infantis 1	2002	0.125	8	8	64	64	2.4 x 10^-11^	64	1
*S.* Livingstone 3	2002	0.25	4	4	16	16	9.9 x 10^-11^	>64	1
*S.* Paratyphi B 5	2004	0.125	2	2	16	16	1.7 x 10^-11^	32	1
*S.* Saintpaul 80969	2008	0.5	16	16	32	32	1.3 x 10^-10^	>64	0.5
*S.* Typhimurium var. Cop.	2008	0.25	2	2	8	8	1.4 x 10^-10^	64	1
*S.* Virchow 2	2002	0.25	1	2	4	8	6.3 x 10^-11^	16	1

^a^ TRC = triclosan

^b^ for determining MPC values, agar plates supplemented with 1 - 128 x the MIC of TRC were used

**Table 2:** Comparison of MIC values and gene expression between *Salmonella* field isolates and their triclosan-selected mutants.

**Strains**	**Mutation in *fabI***	**MIC (µg/ml)^a^**							**x-fold change in gene expression (based on group means)**								
		**TRC**	**GEN**	**KAN**	**CHX**	**CIP**	**TET**	**FLO**	**CHL**		***acrA***	***tolC***	***acrF***	***marA***	***ramA***	***soxS***	***fabI***
*S.* Enteritidis 92		0.25	1	4	2	0.032	1	4	4								
*S.* Enteritidis 92 mutant 2	Gly_93_ – Val	32	0.5	2	1	0.032	1	4	4		1.2 ± 0.04	0.8 ± 0.1	1.1 ± 0.03	2.1 ± 0.5	1.8 ± 0.4	0.5 ± 0.2	1.2 ± 0.3
*S.* Hadar 5		0.125	2	8	2	0.25	32	4	4								
*S.* Hadar 5 mutant 1	Gly_93_ – Val	16	1	4	2	0.25	32	4	4		1.1 ± 0.04	1.1 ± 0.2	0.9 ± 0.3	1.7 ± 0.2	2.8 ± 0.9	0.4 ± 0.1	1.4 ± 0.4
*S.* Infantis 1		0.125	1	4	2	0.25	128	4	4								
*S.* Infantis 1 mutant 4	Gly_93_ – Val	64	0.5	2	1	0.25	128	4	4		1.2 ± 0.2	1.1 ± 0.1	1.2 ± 0.8	1.1 ± 0.2	0.9 ± 0.04	0.5 ± 0.1	1.9 ± 0.3
*S.* Livingstone 3		0.25	2	8	1	0.032	128	4	4								
*S.* Livingstone 3 mutant 2	Gly_93_ – Val	>64	1	4	0.5	0.032	128	4	4		1.5 ± 0.7	0.9 ± 0.02	0.9 ± 0.2	1.4 ± 0.2	1.2 ± 0.1	0.4 ± 0.1	1.3 ± 0.1
*S.* Paratyphi B		0.125	1	4	4	0.125	64	4	4								
*S.* Paratyphi B mutant 1	Gly_93_ – Val	32	0.5	2	2	0.125	64	4	4		0.9 ± 0.1	0.9 ± 0.2	0.9 ± 0.1	2.5 ± 1.1	1.2 ± 0.4	0.6 ± 0.2	12.4 ± 2.1
*S.* Saintpaul 80969		0.5	32	128	8	1	2	8	8								
*S.* Saintpaul 80969 mutant 8	Gly_93_ – Val	>64	16	64	4	1	2	8	8		1.5 ± 0.7	1.01 ± 0.1	0.9 ± 0.3	2.4 ± 0.7	1.1 ± 0.1	0.3 ± 0.1	1.5 ± 0.2
*S.* Typhimurium var. Cop.		0.25	2	8	2	0.125	1	2	2								
*S.* Typhimurium var. Cop. mutant 1	Gly_93_ – Val	64	0.5	4	2	0.125	1	2	2		0.9 ± 0.3	1.1 ± 0.1	0.8 ± 0.1	1.2 ± 0.3	1.1 ± 0.4	1.1 ± 0.2	1.2 ± 0.1
*S.* Virchow 2		0.25	4	8	4	0.25	64	4	256								
*S.* Virchow 2 mutant 1	Gly_93_ – Val	16	1	2	4	0.25	64	4	256		1.9 ± 0.4	1.1 ± 0.1	1.2 ± 0.2	2.1 ± 1.1	3.4 ± 0.4	0.4 ± 0.2	1.5 ± 0.2

^a^ Antimicrobial agents and biocides are abbreviated as follows: CHL = chloramphenicol; CIP = ciprofloxacin; CHX = chlorhexidine; GEN = gentamicin; KAN = kanamycin; TET = tetracycline; TRC = triclosan

### Contribution of *fabI* mutations and *fabI*-overexpression to triclosan tolerance

Comparisons of nucleotide and deduced amino acid sequences of *fabI* genes amplified from parent strains and mutants revealed a single bp exchange at codon 93 (GGT → GTT) that resulted in a Gly_93_ →Val amino acid exchange. This exchange was present in mutants of all eight serovars and previously described to occur in *S. Typhimurium* [20]. In serovar Typhimurium, no correlation of the Gly_93_ →Val amino acid exchange with triclosan MICs could be detected [20]. Therefore, we investigated mutants of various serovars selected on lower concentrations of triclosan (picked from agar plates supplemented with triclosan in a concentration of 1 to 4 dilution steps below the MPC). The amino acid exchange at position Gly_93_ →Val was present in all mutants starting from a concentration of 1 µg/ml triclosan used for selection, suggesting that the emergence of this mutation is an early step in the development of triclosan tolerance. None of the strains possessed a second alteration in *fabI* at positions 115, 159 or 203, taking into account that these positions are less effective in *Salmonella* or *E. coli* in mediating high levels of triclosan tolerance [22,39]. Gene expression analysis of all mutants showed 1.2- to 1.9 -fold increases in *fabI* expression, with the exception of serovar Paratyphi B, demonstrating a 12.4-fold up-regulation of the gene. As previous studies have shown that overexpression of wild-type *fabI* in medium- or high-level triclosan tolerant mutants had either no effect or decreased MIC values of triclosan [20], complementation experiments were performed. As compared with *S. Typhimurium* LT2 and S. Livingstone 4 recipient strains, recipients harbouring wild-type *fabI* on a recombinant vector plasmid exhibited 16-fold increases in MIC values of triclosan, respectively (Table 3). MICs of clones carrying recombinant Gly_93_ →Val *fabI* plasmids were even 512- and 256-fold higher than those of the recipient strains. MIC values of other biocides remained unchanged. Gene expression analysis confirmed 3.7- to 123.3-fold higher levels of *fabI* expression due to the plasmid location of the gene (data not shown). Therefore, it can be assumed that Gly_93_ →Val exchanges and enhanced expression of the *fabI* gene play an important role in the development of triclosan tolerance. Nevertheless, PAβN decreased the MIC_TRC_ values of the clones up to 64-fold, indicating that functional active multiple drug efflux systems are required for higher levels of triclosan tolerance.

**Table 3:** MIC values of triclosan for *E. coli* and *Salmonella* strains harboring *fabI* with and without mutation on a plasmid.

**Bacteria**	***fabI* carriage on plasmids**	**MIC (µg/ml)^a^**					
		**TRC**	**TRC in the presence of 0.25 x MIC PAβN**	**CHL**	**CHL in the presence of 0.25 x MIC PAβN**	**TET**	**TET in the presence of 0.25 x MIC PAβN**
*E. coli* TOP10		0.125	< 0.008	4	1	2	0.5
*E. coli* TOP10::pCR2.1		0.125	< 0.008	4	1	2	0.5
*E. coli* TOP10::pCR2.1	*fabI* original	2	0.125	4	1	2	0.5
*E. coli* TOP10::pCR2.1	*fabI* Gly_93_→Val	64	1	4	1	2	0.5
S. Livingstone 4		0.125	< 0.008	8	2	2	0.5
S. Livingstone 4::pCR2.1	*fabI* original	2	0.5	8	2	2	0.5
S. Livingstone 4::pCR2.1	*fabI* Gly_93_→Val	64	1	8	2	2	0.5
S. Typhimurium LT2		0.25	< 0.008	8	1	1	0.5
S. Typhimurium LT 2::pCR2.1	*fabI* original	4	0.25	8	1	1	0.5
S. Typhimurium LT 2::pCR2.1	*fabI* Gly_93_→Val	64	4	8	1	1	0.5

^a^ CHL = chloramphenicol; TET = tetracycline; TRC = triclosan; PAβN = Phe-Arg-β-naphthylamide

### Increase in aminoglycoside susceptibility and reduced growth rates of triclosan tolerant mutants

Antimicrobial susceptibility testing of triclosan-selected mutants and comparisons to parent strains displayed a decrease in MIC values of the aminoglycoside antibiotics gentamicin and kanamycin, whereas MICs of additionally tested antimicrobials remained the same (Table 2). For the biocides, a 2-fold decrease in MICs occurred in some mutants only for chlorhexidine. These decreases were independent of the classification of the parent strains as susceptible or resistant to gentamicin or kanamycin and comprised 1- or 2- dilution steps in all mutants. Particularly noteworthy is that changes in the susceptibility took place only in mutants cultivated on agar plates supplemented with triclosan, whereas MIC values decreased in line with the parent strains after a few serial passages without triclosan. Albeit minor, the changes could be repeated in three independent test series and macrorestriction analysis confirmed the clonal relationship of the triclosan-adapted strains. A similar observation was made by Cottell et al., who detected a statistically significant increase in the susceptibility of triclosan-selected *E. coli* mutants to gentamicin, amikacin, framycetin, streptomycin and kanamycin compared with parent strains [40]. In *Pseudomonas aeruginosa*, a reduction in tobramycin, amikacin and gentamicin resistance was detected after benzalkonium chloride exposure, but results were strain-dependent [41,42]. In *S. Typhimurium*, changes in outer membrane LPS O-antigen chain molecules have been detected after adaptation to biocide formulations [43]. Whether changes in outer membrane LPS contribute to the MIC reduction of aminoglycosides in triclosan-selected mutants, as suggested in *Pseudomonas* [42], remains to be clarified.

**Figure 1:**
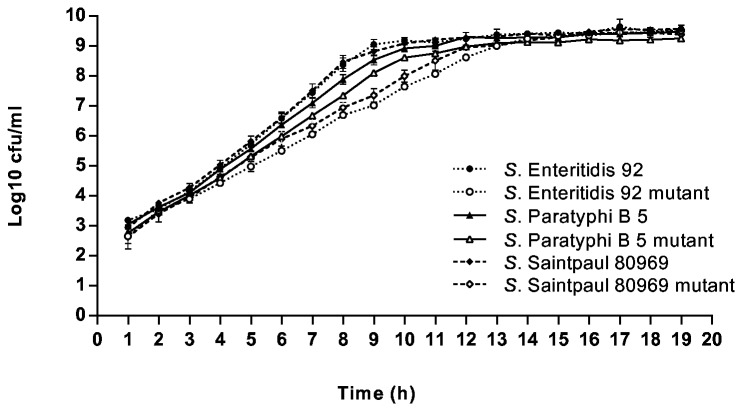
Growth curves of *Salmonella* parent strains and their triclosan-selected mutants. The culture-based enumeration was performed every hour. Each point in the graph represents the average CFU/ml of three independent experiments.

Testing of the phenotypic stability of mutants revealed that elevated MICs of triclosan were not lost in the absence of selective pressure after a daily subculture of generated mutants for 10 successive days. However, the growth rates of mutants were statistically significant lower than for their parent strains, as shown for *S. Enteritidis*, *S*. Paratyphi B and S. Saintpaul mutants (Figure 1). These findings indicate that mutants of various serovars adapted to high concentrations of triclosan are less fit, even if the ability to colonize and persist in the avian gut might be retained, as previously shown for *S. Typhimurium* [20]. Nevertheless, these findings might explain the rare occurrence of decreased susceptibility to triclosan in *Salmonella* field isolates or clinical isolates and the lack of a resistance development, as shown during a comparison of current isolates with isolates collected 1 - 3 decades ago, despite the long-term use of triclosan [21,22].
